# Metabarcoding of Antarctic Lichens from Areas with Different Deglaciation Times Reveals a High Diversity of Lichen-Associated Communities

**DOI:** 10.3390/genes14051019

**Published:** 2023-04-29

**Authors:** Andreas Beck, Angélica Casanova-Katny, Julia Gerasimova

**Affiliations:** 1 SNSB-Botanische Staatssammlung München, 80638 Munich, Germany; 2 GeoBio-Center, Ludwig-Maximilians-Universität München, 80333 Munich, Germany; 3 Laboratorio de Ecofisiología Vegetal y Cambio Climático, Facultad de Recursos Naturales, Universidad Católica de Temuco, Temuco 4780000, Chile

**Keywords:** *Himantormia*, *Placopsis*, *Ramalina*, lichen-associated eukaryotes, deglaciation time

## Abstract

Lichens have developed numerous adaptations to optimise their survival under harsh abiotic stress, colonise different substrates, and reach substantial population sizes and high coverage in ice-free Antarctic areas, benefiting from a symbiotic lifestyle. As lichen thalli represent consortia with an unknown number of participants, it is important to know about the accessory organisms and their relationships with various environmental conditions. To this end, we analysed lichen-associated communities from *Himantormia lugubris*, *Placopsis antarctica*, *P. contortuplicata*, and *Ramalina terebrata*, collected from soils with differing deglaciation times, using a metabarcoding approach. In general, many more Ascomycete taxa are associated with the investigated lichens compared to Basidiomycota. Given our sampling, a consistently higher number of lichen-associated eukaryotes are estimated to be present in areas with deglaciation times of longer than 5000 years compared to more recently deglaciated areas. Thus far, members of Dothideomycetes, Leotiomycetes, and Arthoniomycetes have been restricted to the *Placopsis* specimens from areas with deglaciation times longer than 5000 years. Striking differences between the associated organisms of *R. terebrata* and *H. lugubris* have also been discovered. Thus, a species-specific basidiomycete, *Tremella*, was revealed for *R. terebrata*, as was a member of Capnodiales for *H. lugubris*. Our study provides further understanding of the complex terricolous lichen-associated mycobiome using the metabarcoding approach. It also illustrates the necessity to extend our knowledge of complex lichen symbiosis and further improve the coverage of microbial eukaryotes in DNA barcode libraries, including more extended sampling.

## 1. Introduction

Antarctica is the only continent where the dominant vegetation comprises cryptogamic species. Among these, lichens are the most diverse group, encompassing more than 400 described species [1] that mostly grow on soils or rocks. Thus, they are involved in soil formation processes and nutrient cycling and are key elements of Antarctic terrestrial ecosystems [2,3]. Being poikilohydric, lichens are particularly well-adapted to withstanding adverse environmental conditions and reach large population sizes and high coverage in Antarctic ice-free areas [4,5]. Lichens are a fascinating example of obligate fungal symbiosis and are well known to be composed of at least two main organisms: the fungus (mycobiont) and the algal (photobiont) partner [6,7]. In addition, many more or less closely associated additional players, such as accessory fungi, algae, and bacteria, are known to occur [8,9,10]. Thus, lichen thalli represent not only dual or triple symbiosis but also consortia with an unknown number of participants [11] or complex ecosystems [8,12] comprising lichenicolous and endolichenic fungi, as well as bacterial epi- and endobionts with various potential roles in the whole symbiotic relationship [13].

Until the end of the twentieth century, the taxonomy of lichen-forming fungi and their photobionts was based on morphology, anatomy, and chemistry. However, in recent years, molecular tools, bioinformatics, and growing DNA sequence databases have enabled the study of the inter- and intraspecific diversity of lichen-forming fungi, which harbour additional fungi, algae, and bacteria [14,15,16,17]. The complexity of lichen thalli is part of the reason that lichens are still poorly investigated via omics approaches [15,18]. To reduce problems with chimeric contigs, it is preferred that the sequences of lichen symbionts are obtained by cultivating individual partners axenically [16,17,19], but then, the full diversity of symbiosis is not revealed. Moreover, the isolation of lichen symbionts can be challenging because of the high risk of contamination or the time required for spore discharge (mycobiont). Hence, it might take months to one year before enough material can be obtained for further experiments and sequencing [20,21,22], which makes the metabarcoding and metagenomic approaches beneficial.

Next-generation sequencing studies have discovered lichen-specific bacterial communities and various secondary fungal lineages inhabiting lichens [13,15,23,24]. Several investigators have found basidiomycetous yeasts as symptomless endolichenic fungi on and in lichen thalli [10]. A specific group of basidiomycetous yeasts, Cyphobasidiales (Pucciniomycotina, Basidiomycota), was detected as a component of the lichen microbiome [13]. In addition to a phenotypic relationship, the authors associated the abundance of these basidiomycetous yeasts with the production of lichen secondary substances in *Bryoria fremontii* and *B. tortuosa*. These two aspects were fundamental to their hypothesis of basidiomycetous yeast being the third mutualistic partner within the lichen complex. However, a subsequent broad metagenomic study of 339 lichen species collected from the Appalachian Mountains in North America failed to detect basidiomycete yeasts in over 97% of the sampled species, leading the authors to question their ubiquity in lichens [25]. Next to secondary fungi, concomitant photobionts may alter the physiological properties of lichens. For example, Casano et al. [26] reported two *Trebouxia* algae with different physiological performances, which are always present in the lichen thalli of *Ramalina farinacea*.

The recent application of metabarcoding approaches using high-throughput sequencing (HTS) has highlighted that the microbiome diversity of lichens may be greater than we think (e.g., [27,28,29]) and can help detect cryptic lineages of lichen-associated communities in recently deglaciated soils. Thus, in a recent study, Zhang et al. [30] revealed a high diversity of fungal communities in eleven ice-free Antarctic habitats and elucidated the ecological traits of fungal communities in this unique ice-free area of maritime Antarctica. In addition, Coleine et al. [31] demonstrated the importance of sun exposure on the presence of functional groups of fungi in cryptoendolithic Antarctic communities.

Among the ribosomal cistron regions, the nuclear internal transcribed spacer (nrITS) has been proven to have the most clearly defined barcode gap between inter- and intraspecific variation. For this reason, it was successfully applied in the barcoding of various lichen genera (e.g., [32,33,34,35]) and lichen mycobiomes [36]. Thus, for example, to investigate cryptic diversity in *Parmelia*, Divakar et al. [34] used a DNA barcoding tool for rapid, accurate sample identification and detection and sampled 202 specimens of *Parmelia s.s.* worldwide, including Antarctica, using nrITS. In our study, we used next-generation sequencing to target the nrITS2 region. We used a metabarcoding approach to assess and characterise the diversity of the lichen-associated communities in *Himantormia lugubris*, *Placopsis antarctica*, *P. contortuplicata*, and *R. terebrata*. As the two *Placopsis* species occur in both recently deglaciated and areas that have been ice-free for longer on the South Shetland Islands, we used the genus *Placopsis* to investigate whether or not the associated communities differ between areas with different deglaciation times. Furthermore, specimens of this genus from the volcanic, and thus, warmer Deception Island [Olsacher 1956] were included to compare samples from this peculiar habitat with the ones from the other South Shetland Islands.

## 2. Materials and Methods

### 2.1. Taxon Sampling

**Figure 1:**
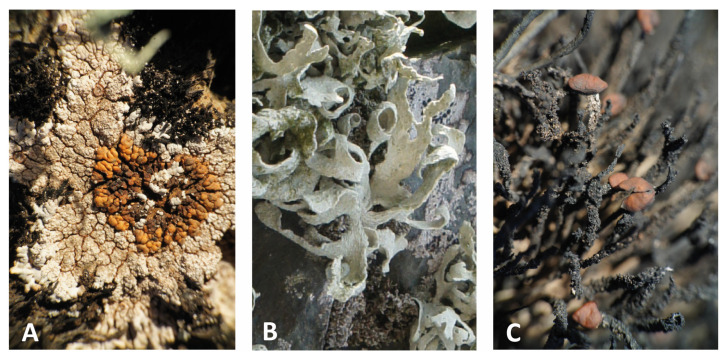
Lichen species investigated in this study: (**A**) *Placopsis antarctica*, (**B**) *Ramalina terebrata*, and (**C**) *Himantormia lugubris*.

Sampling covered different successional stages of lichen communities, from the pioneer phase with the crustose lichens *P. antarctica* D. J. Galloway, R. I. L. Sm. & Quilhot (Figure 1A), and *P. contortuplicata* I. M. Lamb to mature lichen communities, including the fruticose lichens *R. terebrata* Hook. f. & Taylor and *H. lugubris* (Hue) I. M. Lamb (Figure 1B,C). The samples collected were identified using standard identification procedures [1,37], with voucher specimens deposited in the herbarium of SNSB-Botanische Staatssammlung München (M).

This study is based on specimens collected on the South Shetland Islands, Antarctica: Fildes and Potter Peninsula of King George Island, Ardley Island (close to the southwest end of King George Island), Coppermine Peninsula of Roberts Island, Byers Peninsula of Livingston Island, and four localities on Deception Island (Table 1). In total, 32, 10, and 11 thalli of the genus *Placopsis* were included in our study: 28 thalli of *P. antarctica* from long-deglaciation-time regions, seven from short-deglaciation-time regions, and ten from Deception Island, as well as four, three, and one thallus of *P. contortuplicata* from the respective areas. In addition, 14 specimens of *R. terebrata* (7) and *H. lugubris* (7) were included. Due to its unique geology, the deglaciation times of the Deception Island cannot be accurately given.

### 2.2. Sample Preparation, Lysis, and DNA Extraction

From each specimen, ten branch tips 5 mm long from the fruticose lichens *R. terebrata* and *H. lugubris*, or ten areoles of about 0.2 mm^2^ from the crustose lichen *Placopsis*, without any visible contamination of aerophytic organisms, were selected and pooled in 2 mL sterile sample tubes. These tissue samples were subsequently homogenised using a FastPrep96 (MP Biomedicals, Eschwege, Germany), twice for 60 s, with the addition of ceramic beads. Genomic DNA was extracted as described in Beck and Mayr [39], but instead using the Invisorb^®^ Spin Plant Mini Kit (STRATEC Molecular GmbH, Berlin, Germany) according to the manufacturer’s protocol.

### 2.3. DNA Amplification and Metabarcoding

DNA metabarcoding was conducted at the AIM Lab (AIM—Advanced Identification Methods GmbH, Leipzig, Germany). From each sample, 5 µL of extracted total DNA was used for PCR. Plant MyTAQ (Bioline, Luckenwalde, Germany) and high-throughput sequencing (HTS)-adapted mini-barcode primers were used for targeting the Internal Transcribed Sequence 2 (ITS2; primers (gITS7 and ITS4) and amplification following Ihrmark et al. [40]). Amplification success and fragment lengths were verified via gel electrophoresis. Amplified DNA was cleaned using a 1% sodium acetate and 70% ethanol precipitation method and resuspended in 50 µL purified water for each sample before proceeding. Illumina Nextera XT (Illumina Inc., San Diego, CA, USA) indices were ligated to the samples in a second PCR reaction, whereby we applied the same annealing temperature as for the first PCR reaction but with only seven cycles. Ligation success was confirmed via gel electrophoresis. DNA concentrations were measured using a Qubit fluorometer (ThermoFisher Scientific, Waltham, MA, USA) and adjusted to 40 µL pools, each containing equimolar concentrations of 100 ng/µL DNA template. Pools were purified using MagSi-NGSprep Plus beads (Steinbrenner Laborsysteme GmbH, Wiesenbach, Germany) with a final elution volume of 20 µL. High-throughput sequencing (HTS) was performed on an Illumina MiSeq (Illumina Inc., San Diego, CA, USA) using v3 chemistry (2 × 300 paired-end reads, 600 cycles, maximum of 25 million reads).

### 2.4. Barcode Sequence Analysis, Processing, and Amplicon Sequencing Variant (ASV) Identification

Sequence processing was performed with the VSEARCH v2.4.3 suite [41] and cutadapt v1.1439 [42]. Since not all of the sequenced samples yielded reverse reads of high enough quality to enable paired-end merging, only forward reads were utilised. Reads were removed in cases where the number of mismatches was >5, the alignment was shorter than 16 base pairs, or the identity percentage of the alignment was <90%. Forward primers and adaptors were removed with cutadapt [42]. Quality filtering was performed with the fastq_filter program in VSEARCH [41] (fastq_maxee 2, minimum length of 100 bp). Subsequently, sequences were dereplicated using VSEARCH [41] with derep_fulllength, first at the sample level, then concatenated into one fasta file, which was then dereplicated. Chimeric sequences were filtered from the large fasta file with VSEARCH [41] using uchime_denovo. The remaining sequences were clustered into amplicon sequencing variants (ASVs) at 97% identity with cluster_size, and an ASV table was created using VSEARCH [41] with usearch_global. To reduce false positives, a cleaning step was employed, which excluded read counts in the ASV table of less than 0.01% of the total. ASVs were blasted against a custom database downloaded from UNITE (February 2020), NCBI GenBank (January 2022), and BOLD (October 2021), including taxonomy and barcode index number (BIN) information (contained in the BOLD database), using Geneious (v.10.2.5; Biomatters, Auckland, New Zealand) and following the methods described by Moriniere et al. [43]. The resulting csv file, which included the ASV ID, BOLD process ID, BIN, Hit-%-ID value (percentage of overlap similarity, i.e., identical base pairs, of an ASV query sequence with its closest counterpart in the database), length of the top BLAST hit sequence, and phylum, class, order, family, genus, and species information for each detected ASV, was exported from Geneious and combined with the ASV table generated by the bioinformatic pipeline (Supplementary Data S1). The taxonomy of the most complete UNITE BLAST was used in subsequent analyses (Supplementary Data S1).

### 2.5. Sample Pooling, Data Exclusion, and Plausibility Control

Of the 429 ASVs retrieved, 359 ASVs could be identified at the genus level or below via metabarcoding (Supplementary Data Table S1). Obvious contaminants known not to occur in Antarctica were excluded from the analysis. Data on associated eukaryote composition were compiled and analysed, focussing on presence/absence because metabarcoding does not allow for accurate estimations of symbiont quantity [43,44]. Relative read percentages are thus intended to provide rough estimates.

### 2.6. KRONA Metagenomic Visualisation

Interactive HTML charts were created using KronaTools v2.7 [45] (https://github.com/marbl/Krona/wiki/KronaTools, accessed on 20 April 2021). Three sets of charts were created based on ASV table taxonomic annotations from NCBI, UNITE, and BOLD. In each case, a set of charts was created for each sample and for reads summed over all samples. Read counts of different ASVs annotated to the same species were combined in each chart. We used a custom script to extract the ASV counts and associated taxonomic annotations from the final Excel results table. Then, we created the intermediate sample count (.TAX) files for KronaTools using a bash script obtained from https://github.com/GenomicaMicrob/OTUsamples2krona (accessed on 20 April 2021), where the charts were created using the command (ktImportText [SAMPLE.TAX] -n SAMPLE -o SAMPLE.html). The snapshot function of the HTML Krona charts was used to export figures that showed the relevant organism distribution.

### 2.7. Diversity Estimations

To estimate the expected diversity in the three communities, coverage and sample-size-based rarefaction/extrapolation curves were computed using iNEXT [46]. Curves with 95% confidence intervals were calculated for Hill numbers q = 0 (species richness), q = 1 (the exponential of Shannon’s entropy index), and q = 2 (the inverse of Simpson’s concentration index). The size in the rarefaction/extrapolation curve was extrapolated to triple the minimum observed sample size, guided by an estimated asymptote (endpoint = 32), as recommended by Chao et al. [47,48].

The alpha diversity was calculated using the diversity function implemented in the vegan package [49] based on the number and relative abundance of taxa (species, or ASVs) to quantify the diversity in each of the three communities. The outliers were subsequently removed from this analysis (i.e., those whose alpha index was less than 0.4 and higher than 1.6). To test if at least one of the diversity means was different from the others, an analysis of variance (ANOVA) in the vegan package was employed [49]. Subsequently, the pairwise comparisons implemented in the honest significant difference test function (HSD.test) from the agricolae package [50] were used to analyse whether a set of communities’ means were different from each other. All graphics were created using the ggplot2 R package [51]. The scripts for coverage, sample-size-based rarefaction/extrapolation curves and alpha-diversity calculations are provided in the Supplementary Materials Scripts.

## 3. Results and Discussion

### 3.1. Eukaryotes Associated with the Lichens Himantormia, Placopsis, and Ramalina

**Figure 2:**
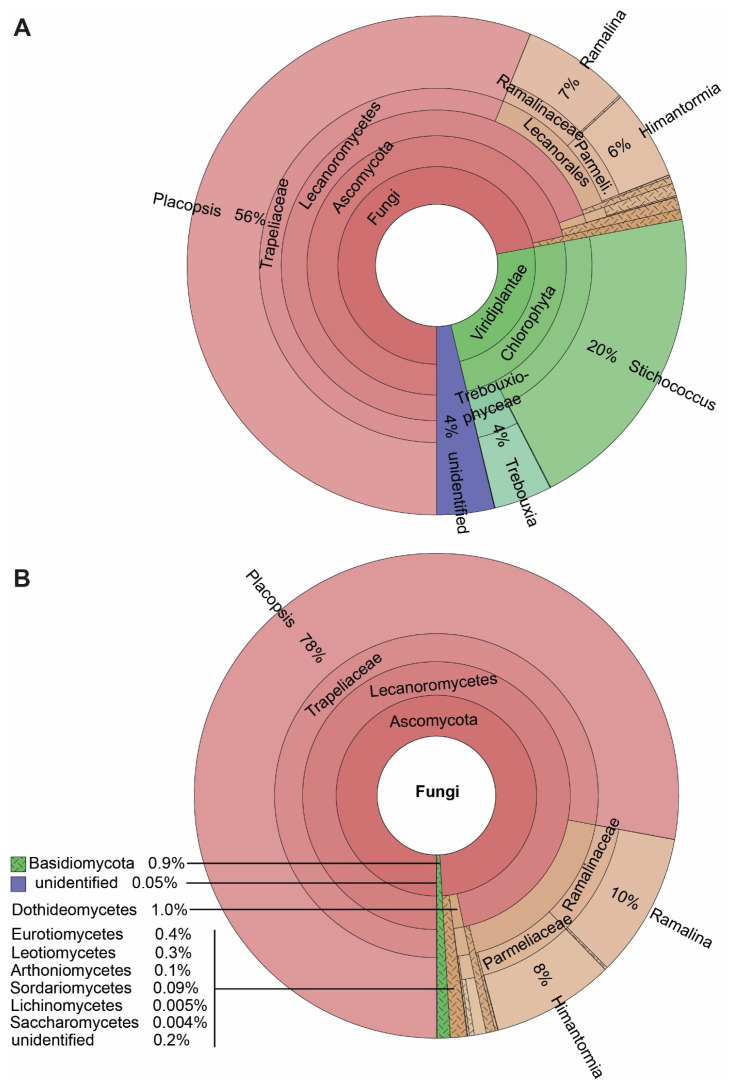
The spectrum of taxa associated with the four Antarctic lichens *Himantormia lugubris*, *Placopsis antarctica*, *P. contortuplicata*, and *Ramalina terebrata*. (**A**) All eukaryotes. (**B**) Fungi.

Our results reveal a large number of lichen-associated organisms. Most reads expectedly corresponded to the mycobionts of the four lichens investigated, viz. *P. antarctica*, *P. contortuplicata*, *R. terebrata*, and *H. lugubris*, members of the class Lecanoromycetes. Given that *P. contortuplicata* is represented by a few specimens, we united the data for both *Placopsis* species in the Krona plots. The major photobionts of these lichens, *Stichococcus* in the two *Placopsis* species and *Trebouxia* in *Ramalina* and *Himantormia* comprised the next most abundant ASVs. Only 4% of the ASVs remained without a determined close match, primarily due to an unidentified *Trebouxia* symbiont of *Himantormia* (ASV_6; 2.8%) assigned to this genus in the NCBI search. The Ascomycota and Basidiomycota ASVs from different classes were recovered, namely from Dothideomycetes, Eurotiomycetes, Leotiomycetes, Arthoniomycetes, Sordariomycetes, Lichenomycetes, Saccharomycetes, Agaricomycetes, and Tremellomycetes, respectively. Given this large spectrum of coverage, the presented metabarcoding method is well suited to analysing lichen-associated eukaryotes, with an emphasis on lichen-associated fungi, as they comprise the largest group based on the number of reads and the number of ASVs (Figure 2).

### 3.2. ASVs Associated with the Fruticose Lichens Ramalina terebrata and Himantormia lugubris

**Figure 3:**
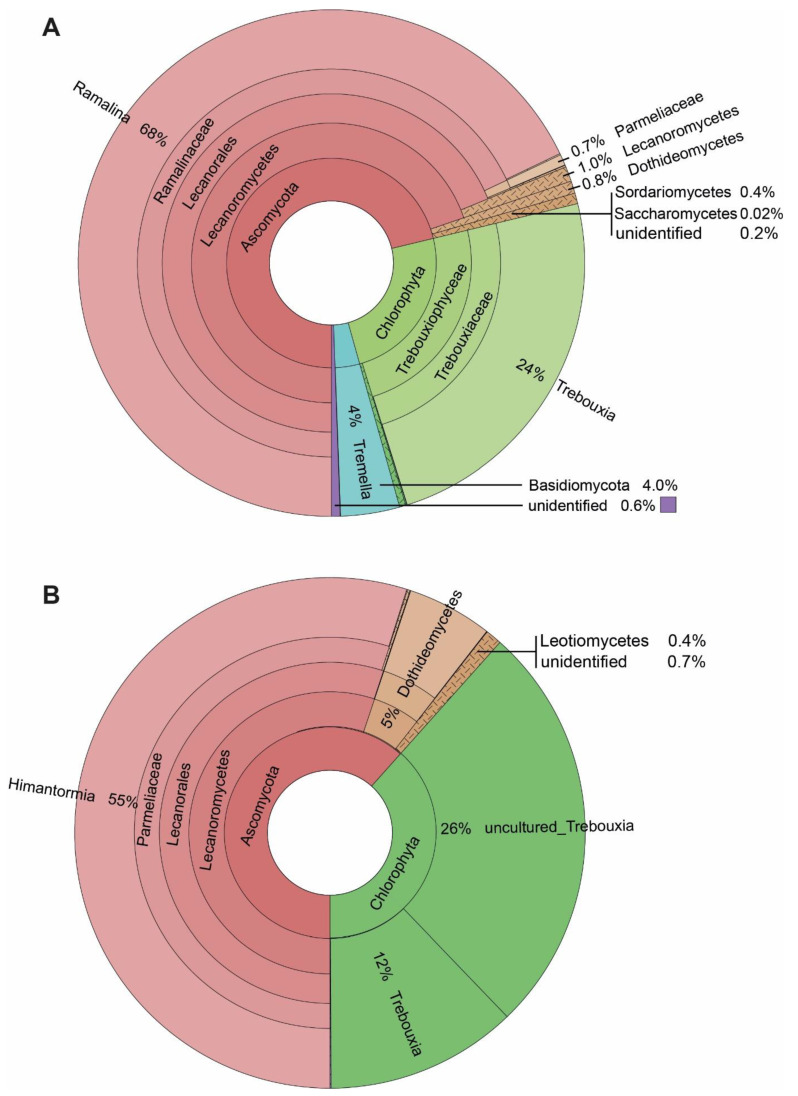
The spectrum of taxa associated with the Antarctic lichens: (**A**) *Ramalina terebrata*, and (**B**) *Himantormia lugubris*.

Our results show 168 ASVs associated with the seven specimens of *R. terebrata* (Figure 3). Most reads were related to the mycobiont (68%). Next to the major photobiont (*Trebouxia*, comprising 98% of all Chlorophyta reads), the algal genera *Stichococcus* (1%), as well as *Coccomyxa*, *Desmococcus*, *Prasioloa*, and *Symbiochloris*, were recorded in very small amounts (Supplementary Table S1). Associated basidiomycete fungi were present in relatively high amounts, in addition to the ascomycete fungi. Four percent of the reads belonged to the genus *Tremella*, and members of the recently described lichen-associated basidiomycete yeasts, the Cystobasidiomycetes (0.3%; four out of seven specimens; Supplementary Table S1). The associated Ascomycetes comprised only about one-third of the reads assigned to Basidiomycetes (Figure 3) and belonged to the classes Dothideomycetes (mainly Capnodiales) and Sordariomycetes (mainly Hypochreales).

Compared to the rich spectrum of lichen-associated organisms in *R. terebrata*, only about one-third (54) ASVs, were recorded for the seven specimens of *H. lugubris*. Again, most reads belonged to the mycobiont (55%), and for algae, almost only the major photobiont, *Trebouxia*, was recovered. The relatively low number of mycobiont reads in *H. lugubris* compared to *R. terebrata* (55% versus 68%) may be due to its massive central chondroid axis with thick cell walls [52], resulting in comparatively fewer cells per sample volume, and consequently, lower DNA content. *Stichococcus* (0.03%), as well as *Coccomyxa* and *Desmococcus* (0.02% each), were also recovered, but in tiny amounts (Supplementary Table S1). In addition to the primary symbionts, most reads were assigned to *Parateratosphaeria marasasii* (Capnodiales, Dothideomycetes; ASV_10), but with 80.4% identity only (Supplementary Table S1). Thus, this taxon, identified in all seven *Himantormia* specimens, was sequenced for the first time. The next most abundant ASVs, *Gorgomyces* and *Chalara* (Leotiomycetes), were recorded in only two of the seven specimens.

Taken together, the most striking difference between the associated organisms in *R. terebrata* and *H. lugubris* was the exclusive occurrence of Basidiomycetes in *R. terebrata*. On the other hand, taxon ASV_10 from Dothideomycetes, whose nrITS2 sequence was most similar to that of *P. marasasii* (Capnodiales), was the most frequently associated organism and present in all seven *Himantormia* specimens. At the same time, it was detected only in tiny amounts in one *Ramalina* specimen.

### 3.3. ASVs Associated with the Crustose Lichen Genus Placopsis Growing on Soils with Different Deglaciation Times

**Figure 4:**
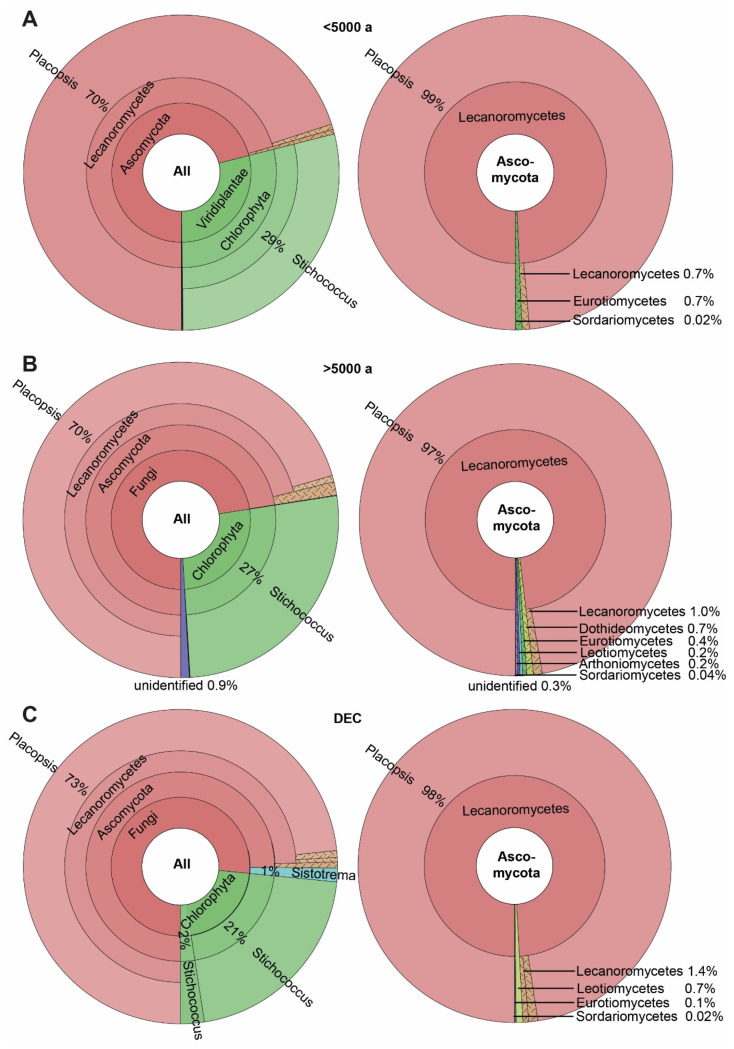
The spectrum of taxa associated with the Antarctic lichen *Placopsis* growing on soils with different deglaciation times. (**A**) Deglaciated less than 5000 years ago, (**B**) deglaciated more than 5000 years ago, and (**C**) volcanic soil of Deception Island. The Krona plots on the **left** depict the distribution of all taxa, and on the **right** indicate Ascomycota only.

A total of 129 ASVs were detected in the 53 *Placopsis* specimens. These specimens were separated into three groups: two differing in deglaciation time and one from the volcanic Deception Island (due to its unique geology, the deglaciation times of the island cannot be accurately given). The specimens collected from soils that have been deglaciated for longer than 5000 years contained 122 of these ASVs, and those collected from Deception Island comprised 75 ASVs. The specimens collected from soils that were deglaciated less than 5000 years ago had 60 ASVs (Supplementary Table S1). These numbers were caused by the higher number of associated fungi growing on soils with longer deglaciation times. Members of the Dothideomycetes, Leotiomycetes, and Arthoniomycetes were restricted to the lichen specimens from soils that have been deglaciated for longer (Figure 4).

**Figure 5:**
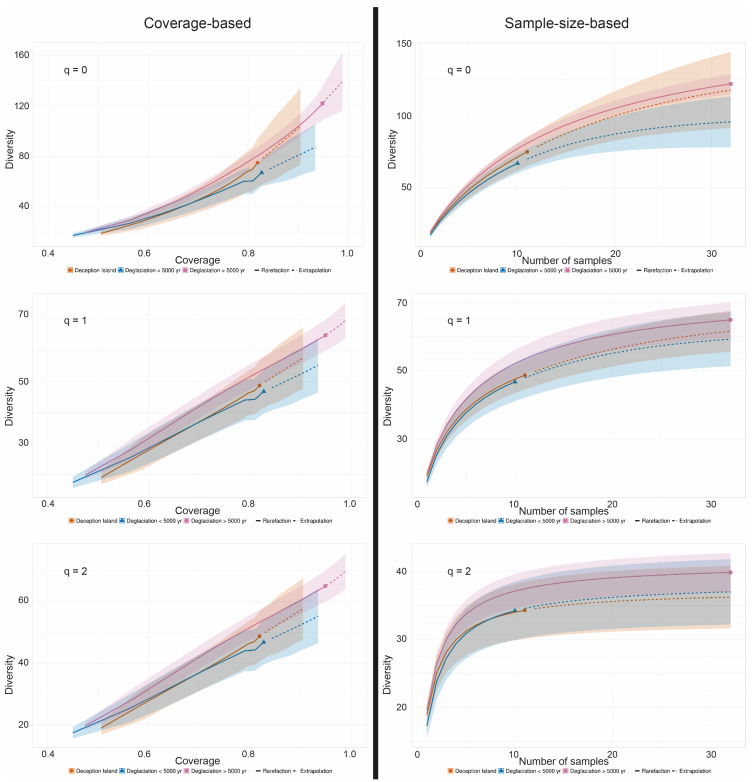
A comparison of coverage- (**left** side) and sample-size-based (**right** side) rarefaction (solid line) and extrapolation (dotted line) curves with 95% confidence intervals for Hill numbers q = 0 (species richness—**upper** panel), q = 1 (the exponential of Shannon’s entropy index—**middle** panel), and q = 2 (the inverse of Simpson’s concentration index—**lower** panel) for taxa associated with the Antarctic lichen genus *Placopsis* growing on soils with different deglaciation times. Sample-size-based extrapolation was done up to the largest sample size, i.e. 32, guided by an estimated asymptote. Solid symbols denote reference samples. Samples collected from the volcanic soil of Deception Island (circles, orange colour), soils deglaciated less than 5000 years ago (triangles, blue colour), and soils deglaciated more than 5000 years ago (squares, purple colour).

To account for the uneven sample number between the three communities (namely, that with more than 5000 years of deglaciation time, that with less than 5000 years deglaciation time, and that from the volcanic island Deception Island (32, 10, and 11 specimens, respectively), coverage-based and sample-size-based rarefaction/extrapolation curves were calculated using iNEXT [46] for a base sample size of 32, as recommended by Chao et al. [47] (Figure 5). Consistently, the estimate of ASV diversity (q = 0) was lowest for the samples from areas with a deglaciation time of less than 5000 years, followed by Deception Island, with the highest species number occurring in the regions with deglaciation times of longer than 5000 years. The 95% confidence intervals did not overlap for the samples from areas with different deglaciation times using the sample-size-based approach, indicating a significantly higher species number in the communities with longer deglaciation times. The species number estimates for the rarefied and extrapolated numbers of species were 100, 135, and 145, respectively; thus, the species number of lichen-associated ASVs was estimated to be about 50% higher on soils that have been deglaciated for more than 5000 years compared to those from soils that were deglaciated less than 5000 years. The extrapolation curves of less sampled communities tend to grow over the actual sampling size, which indicates that more comprehensive data are necessary to corroborate these results.

**Figure 6:**
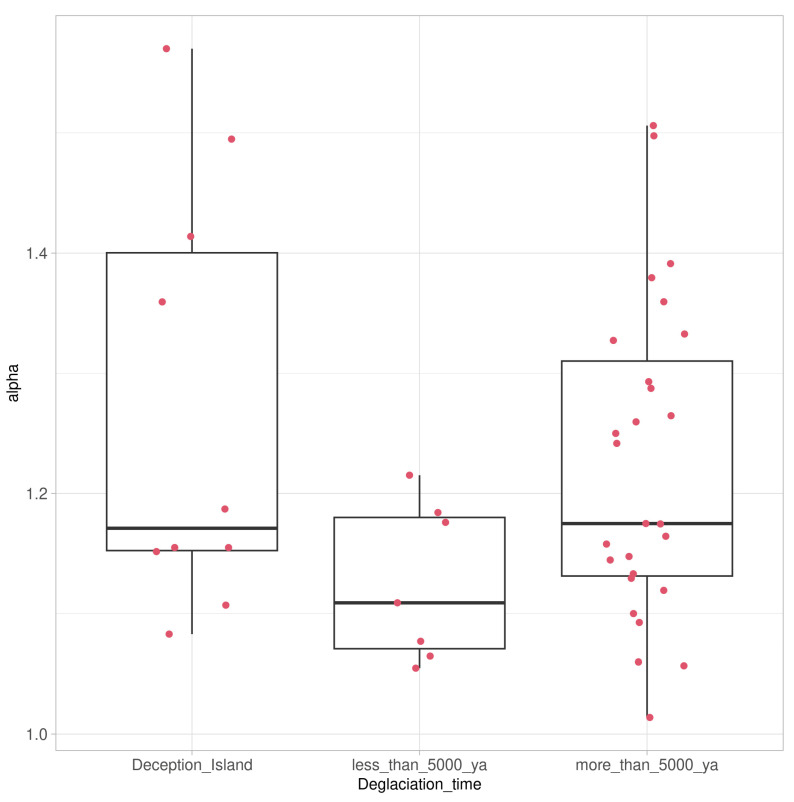
Box-and-whisker plot comparing alpha diversity of lichen-associated communities as expressed by the Shannon Index for taxa associated with the Antarctic lichen genus *Placopsis* growing on soils with different deglaciation times. Samples collected from the volcanic soil of Deception Island (**left**), soils deglaciated less than 5000 years ago (**middle**), and soils deglaciated more than 5000 years ago (**right**). Box-plot indicates median and lower and upper quartiles, with whiskers drawn within the 1.5 IQR; red dots indicate individual values.

The alpha diversity of lichen-associated communities as expressed by the Shannon Index was also highest for the samples from soils with longer deglaciation times, followed by those collected on Deception Island, and was lowest in samples taken from soils with deglaciation times of less than 5000 years (Figure 6). Despite these differences, the HSD test showed that the alpha diversity of all three communities did not differ significantly, assigning them to one group.

Given the not-yet-complete sampling (see above) we only mention some interesting ASVs and give the currently known distribution pattern only for those taxa present in more than 20% of the samples from a given origin, as a guidance for further studies. Concerning the associated algae (i.e., not the main green algal symbiont), ten different ASVs of the genus *Trebouxia* were present in quite a low abundance, irrespective of the deglaciation time. Of these, *Trebouxia* sp. ‘A04’ (Trebouxiales, Chlorophyta; ASV_197) was unique to Deception Island and was present in three out of the eleven specimens. The Antarctic snow alga *Chlorominima collina* [53] was found in two lichen specimens from more recently deglaciated soils, while it occurred in only one specimen from soils with a longer deglaciation history. It did not appear in the specimens from Deception Island. In contrast, the genera *Desmococcus* and *Symbiochloris* were only present in soil specimens with a longer deglaciation time. The ASVs closely related to *Stichococcus allas* were mainly found in specimens from Deception Island (five out of eleven), while it occurred in only 10% of the specimens from the other South Shetland Islands.

Intriguingly, the Basidiomycete *Sistotrema autumnale* was found only in specimens from Deception Island (three out of eleven) and in one specimen from the areas that have been deglaciated for longer than 5000 years (Figure 4). This organism is already known from a soil sample in Antarctica (Unite: UDB07059034; [54]) and as a lichenicolous fungus from *Physcia aipolia* (Unite: MN902070; [55]). Dothideomycetes were exclusively found in areas that have been deglaciated for longer than 5000 years. A taxon related to the genus *Bryochiton* (Capnodiales; ASV_14) was the most frequent in seven out of the thirty-two specimens. However, its closest relative, *B. perpusillus*, had only 89.5% sequence identity and, thus, clearly represented a distinct species based on sequence identity. The Arthoniomycetes were exclusively found in areas that have been deglaciated for over 5000 years. Of these, the genus *Bryostigma* is known to contain lichenicolous species [56].

A possible member of Herpotrichiellaceae (Chaetothyriales, Eurotiomycetes; ASV_207) was found to be unique to areas deglaciated less than 5000 years ago and was present in two out of the ten specimens, with 83.3% identity in the NCBI BLAST search. The next closest hit showed 82.4% identity to Dothideomycetes, but this is most likely due to a misidentification, as the next similar GenBank hits referred to Chaetothyriales as well. Thus, ASV_207 is most likely a member of Chaetothyriales, with no close relative sequenced so far. Moreover, the specimens from Deception Island contained a unique ASV related to a member of the genus *Tetracladium* sp. (Helotiales, Leotiomycetes; ASV_119). It was found to be present in three out of the eleven specimens.

## 4. Conclusions

Our study is the first to employ a DNA metabarcoding approach to lichens growing on soils with differing deglaciation times collected in Antarctica. This is a baseline study, that revealed the first distribution patterns of lichen-associated eukaryotes. However, given the small sample size of communities with short deglaciation times, the results still need further corroboration by extending this sample size. Nevertheless, some patterns already emerge: Overall, there are many more Ascomycete taxa associated with lichens compared to Basidiomycota. Significantly more lichen-associated eukaryotes are estimated to be present in areas with deglaciation times of longer than 5000 years compared to more recently deglaciated areas (Figure 5). The occurrence of several lichen-associated organisms seems to be related to the deglaciation time of their substrate. An intriguing example is a member of the genus *Bryochiton* (Capnodiales), which was found exclusively in 20% of the *Placopsis* growing in ice-free areas with deglaciation times of longer than 5000 years, while a member of the Herpotrichiellaceae (Chaetothyriales) was unique to areas deglaciated less than 5000 years ago. As for Basidiomycetes, lichenicolous *Tremella* and Cystobasidiomycetes seem restricted to *R. terebrata*. Some taxa are found almost exclusively in one of the lichens studied and are present in all specimens analysed. Interesting examples are ASV_16, which is related to the *Tremella* genus and occurs almost exclusively in *R. terebrata*, and ASV_10, which is distantly related to *P. marasasii* (Dothideomycetes) and occurs almost exclusively in *H. lugubris*. 

Although ten different parts of the lichen specimens were pooled to account for heterogeneity (viz., thallus tips and areoles, respectively), considerable variation in the associated organisms can still be observed. Thus, the Cystobasidiomycete representatives were only detected in about half of the specimens, possibly due to the patchy distribution or varying quantities of these associated eukaryotes. These results are in line with those obtained by Dal Grande et al. [57] and Rolshausen et al. [58], where the importance of environmental factors and host identity on the community structure of green algal symbionts and lichen holobionts was shown. Taken together, the environment seems to be the most crucial factor for the formation of lichen holobionts.

The ongoing growth of DNA barcode libraries, such as NCBI and UNITE, with the regular addition of new reference barcodes, will increase the capability of this metabarcoding approach in the future. Although many barcoding efforts have been undertaken in recent years (see Introduction), microbial eukaryotes are still not well covered. This fact can be exemplified by ASV_207, most closely related to Herpotrichiellaceae (Chaetothyriales, Eurotiomycetes), but with only 83.8% identity. In addition, caution still needs to be taken when interpreting results from one database alone. This can be exemplified by ASV_207 being assigned to different classes of Ascomycetes: to Dothideomycetes in NCBI BLAST (identity 84.2%) and Eurotiomycetes in the UNITE database (identity 83.8%). However, the BLAST result is most likely erroneneous, as the next similar GenBank hits also refer to Chaetothyriales. Thus, ASV_207 seems to be a member of Chaetothyriales, whose close close relative has not been sequenced so far. In the context of climate change, not only are temperatures expected to rise but also large areas of the previously ice-covered land surface are expected to be exposed, estimated to be up to 25% of the current ice-free area [59]. Connectivity between the previously relatively isolated ice-free patches will be increased, leading to an easier exchange of organisms, with an anticipated effect on biodiversity [60]. Consequently, it is essential to have baseline information on terrestrial biodiversity, as shown in this study. Yet, a significant fraction of this biodiversity is still not well analysed, if at all. This is, however, very important, as the successional dynamics of such communities may differ, as has recently been shown for bacterial and fungal communities in recently deglaciated soils of the maritime Antarctic [61]. In addition, it has been demonstrated that lichen-associated eukaryotes may differ between seasons [62]. In Antarctica, the seasons are significantly pronounced and considerably differ, e.g., increased snow cover and sub-zero temperatures. Still, it has been shown that lichens can perform photosynthesis below the snow, and even in a frozen state [63,64]. Thus, seasonal dynamics of lichen-associated organisms may occur even under the harsh conditions in Antarctica, which should be studied in detail in the future.

## Figures and Tables

**Table 1:** Voucher information for lichen specimens used in this study. Locality abbreviations: Ar = Ardley, By = Byers Peninsula, Co = Coppermine Peninsula, DI = Deception Island, FI = Fildes Peninsula, KGI = King George Island, LI = Livingston Island, Po = Potter Peninsula, RI = Roberts Island; C = centre, E = east, NE = northeast, NW = northwest, S = south, SE = southeast, SW = southwest, W = west. Deglaciation time was given by Beck et al. [38].

Taxa Used in This Study	Voucher Information	Deglaciation Time	Herbarium Number/Collection Number	Sample Number
*Himantormia* *lugubris*	Antarctica, King George Island, Ardley. S 62°12.622′, W 58°55.848′; 10 m asl.	>5000 a	M-0019715/140116b	B1583
*H.* *lugubris*	Antarctica, King George Island, Fildes Peninsula, Co Gemelos. S 62°11.780′, W 58°59.602′; 33 m asl.	>5000 a	M-0019714/140111	B1582
*H.* *lugubris*	Antarctica, King George Island, Fildes Peninsula, Meseta La Cruz. S 62°12.360′, W 58°57.239′; 49 m asl.	>5000 a	M-0019719/140109	B1587
*H.* *lugubris*	Antarctica, King George Island, Fildes Peninsula, Meseta La Cruz. S 62°12.360′, W 58°57.239′; 49 m asl.	>5000 a	M-0019708	B1666
*H.* *lugubris*	Antarctica, King George Island, Fildes Peninsula, Meseta La Cruz. S 62°12.360′, W 58°57.239′; 49 m asl.	>5000 a	M-0019710/140109	B1668
*H.* *lugubris*	Antarctica, Livingston Island, Byers Peninsula. S 62°38.345′, W 61°05.380′; 94 m asl./LI_By-C	<5000 a	M-0019716/140129c	B1584
*H.* *lugubris*	Antarctica, Livingston Island, Byers Peninsula. S 62°38.345′, W 61°05.380′; 94 m asl./LI_By-C	<5000 a	M-0019711	B1669
*Placopsis antarctica*	Antarctica, Deception Island. S 62°59.211′, W 60°40.534′; 100 m asl./DI-SW		M-0019706/ Dec 15/05aI	B1664
*P. antarctica*	Antarctica, Deception Island. S 62°59.211′, W 60°40.534′; 100 m asl./DI-SW		M-0019704/ Dec 15/05aVI	B1661
*P. antarctica*	Antarctica, Deception Island. S 62°59.211′, W 60°40.534′; 100 m asl./DI-SW		M-0019673/ Dec 15/05cV	B1618
*P. antarctica*	Antarctica, Deception Island. S 62°57.945′, W 60°42.912′; 20 m asl./DI-NW		M-0019700/ Dec 15/07aIV	B1645
*P. antarctica*	Antarctica, Deception Island. S 62°57.945′, W 60°42.912′; 20 m asl./DI-NW		M-0019701/ Dec 15/07bV	B1646
*P. antarctica*	Antarctica, Deception Island. S 62°57.945′, W 60°42.912′; 20 m asl./DI-NW		M-0019664/ Dec 15/07cII	B1609
*P. antarctica*	Antarctica, Deception Island. S 62°56.330′, W 60°35.648′; 30 m asl./DI-NE		M-0019698/ Dec 15/08bII	B1643
*P. antarctica*	Antarctica, Deception Island. S 62°56.330′, W 60°35.648′; 30 m asl./DI-NE		M-0019699/ Dec 15/08cI	B1644
*P. antarctica*	Antarctica, Deception Island. S 62°59.309′, W 60°32.915′; 120 m asl./DI-SE		M-0019672/ DEC 15/04bII	B1617
*P. antarctica*	Antarctica, Deception Island. S 62°59.312′, W 60°32.928′; 115 m asl./DI-SE		M-0019730/ 150202a4_DecWB	B1598
*P. antarctica*	Antarctica, King George Island, Ardley. S 62°12.645′, W 58°56.139′; 33 m asl./KGI-Ar	>5000 a	M-0019686/ ARD 15/03bV	B1631
*P. antarctica*	Antarctica, King George Island, Ardley. S 62°12.645′, W 58°56.139′; 33 m asl./KGI-Ar	>5000 a	M-0019687/ ARD 15/03aIII	B1632
*P. antarctica*	Antarctica, King George Island, Ardley. S 62°12.645′, W 58°56.139′; 33 m asl./KGI-Ar	>5000 a	M-0019655/ ARD 15/03bVI	B1600
*P. antarctica*	Antarctica, King George Island, Fildes Peninsula, Co Gemelos. S 62°11.780′, W 58°59.602′; 33 m asl./KGI-Fi-C	>5000 a	M-0019685/ LGEM 15/01aIII	B1630
*P. antarctica*	Antarctica, King George Island, Fildes Peninsula, Co Gemelos. S 62°11.780′, W 58°59.602′; 33 m asl./KGI-Fi-C	>5000 a	M-0019658/ LGEM 15/01cVII	B1603
*P. antarctica*	Antarctica, King George Island, Fildes Peninsula, Co Gemelos. S 62°11.780′, W 58°59.602′; 33 m asl./KGI-Fi-C	>5000 a	M-0019659/ LGEM 15/01dIV	B1604
*P. antarctica*	Antarctica, King George Island, Fildes Peninsula, Collins/Green Point. S 62°10.164′, W 58°51.373′; 79 m asl./KGI-Fi-NE	>5000 a	M-0019693/ 140118d_GreP	B1638
*P. antarctica*	Antarctica, King George Island, Fildes Peninsula. S 62°13.382′, W 59°01.137′; 49 m asl./KGI-Fi-S	>5000 a	M-0019689/ FiSo 15/01bIII	B1634
*P. antarctica*	Antarctica, King George Island, Fildes Peninsula. S 62°13.382′, W 59°01.137′; 49 m asl./KGI-Fi-S	>5000 a	M-0019690/ FiSo 15/01dIV	B1635
*P. antarctica*	Antarctica, King George Island, Fildes Peninsula. S 62°13.382′, W 59°01.137′; 49 m asl./KGI-Fi-S	>5000 a	M-0019663/ FiSo 15/01aV	B1608
*P. antarctica*	Antarctica, King George Island, Fildes Peninsula, Meseta La Cruz. S 62°12.360′, W 58°57.239′; 49 m asl./KGI-Fi-C	>5000 a	M-0019656/ MCRU 15/01aII	B1601
*P. antarctica*	Antarctica, King George Island, Fildes Peninsula. S 62°10.026′, W 58°56.667′; 42 m asl./KGI-Fi-NW	<5000 a	M-0019688/ 140115a_VALK	B1633
*P. antarctica*	Antarctica, King George Island, Fildes Peninsula. S 62°12.811′, W 59°00.567′; 197 m asl./KGI-Fi-S	>5000 a	M-0019676/ 140121b_ProSch	B1621
*P. antarctica*	Antarctica, King George Island, Fildes Peninsula. S 62°13.733′, W 58°58.385′; 47 m asl./KGI-Fi-S	>5000 a	M-0019682/ FiSo 15/03fVI	B1627
*P. antarctica*	Antarctica, King George Island, Fildes Peninsula. S 62°13.733′, W 58°58.385′; 47 m asl./KGI-Fi-S	>5000 a	M-0019691/ FiSo 15/03cVI	B1636
*P. antarctica*	Antarctica, King George Island, Fildes Peninsula. S 62°13.733′, W 58°58.385′; 47 m asl./KGI-Fi-S	>5000 a	M-0019692/ FiSo 15/03eI	B1637
*P. antarctica*	Antarctica, King George Island, Fildes Peninsula. S 62°13.733′, W 58°58.385′; 47 m asl./KGI-Fi-S	>5000 a	M-0019665/ FiSo 15/03bV	B1610
*P. antarctica*	Antarctica, King George Island, Potter Peninsula. S 62°14.887′, W 58°40.378′; 68 m asl./KGI-Po	>5000 a	M-0019675/ 150117c_Potter	B1620
*P. antarctica*	Antarctica, King George Island. Northern part of Fildes Peninsula. S 62°8.727′, W 58°54.341′; 45 m asl./KGI-Fi-NW	<5000 a	M-0019684/ VALK 15/02dII	B1629
*P. antarctica*	Antarctica, King George Island. Northern part of Fildes Peninsula. S 62°8.727′, W 58°54.341′; 45 m asl./KGI-Fi-NW	<5000 a	M-0019661/ VALK 15/02bII	B1606
*P. antarctica*	Antarctica, King George Island. Northern part of Fildes Peninsula, Green Point. S 62°10.165′, W 58°51.294′; 50 m asl./KGI-Fi-NE	<5000 a	M-0019666/ GreP 15/01eIV	B1611
*P. antarctica*	Antarctica, King George Island. Northern part of Fildes Peninsula, Green Point. S 62°10.165′, W 58°51.294′; 50 m asl./KGI-Fi-NE	<5000 a	M-0019667/ GreP 15/01dI	B1612
*P. antarctica*	Antarctica, King George Island. Northern part of Fildes Peninsula. S 62°10.628′, W 58°58.048′; 50 m asl./KGI-Fi-C	>5000 a	M-0019662/ VALK 15/04bI	B1607
*P. antarctica*	Antarctica, King George Island. Potter Peninsula. S 62°14.570′, W 58°39.675′; 64 m asl./KGI-Po	>5000 a	M-0019694/ Carl 15/01cI	B1639
*P. antarctica*	Antarctica, King George Island. Potter Peninsula. S 62°14.570′, W 58°39.675′; 64 m asl./KGI-Po	>5000 a	M-0019695/ Carl 15/01eII	B1640
*P. antarctica*	Antarctica, King George Island. Potter Peninsula. S 62°14.570′, W 58°39.675′; 64 m asl./KGI-Po	>5000 a	M-0019668/ Carl 15/01aI	B1613
*P. antarctica*	Antarctica, Livingston Island, Byers Peninsula. S 62°39.210′, W 61°00.444′; 60 m asl./LI_By-E	<5000 a	M-0019678/ 140128e_CeNegro	B1623
*P. antarctica*	Antarctica, Livingston Island, Byers Peninsula. S 62°38.345′, W 61°05.380′; 94 m asl./LI_By-C	<5000 a	M-0019731/ 140129c_CeChester	B1599
*P. antarctica*	Antarctica, Livingston Island, Byers Peninsula, Cerro Smellie. S 62°39.112′, W 61°08.761′; 15 m asl./LI-By-W	>5000 a	M-0019696/ 140130c_CeSmellie	B1641
*P. antarctica*	Antarctica, Livingston Island, Byers Peninsula. S 62°40.065′, W 60°54.739′; 130 m asl./LI-By-E	>5000 a	M-0019697/ Byer 15/06bIII	B1642
*P. antarctica*	Antarctica, Livingston Island, Byers Peninsula. S 62°40.065′, W 60°54.739′; 130 m asl./LI-By-E	>5000 a	M-0019671/ Byer 15/06dIII	B1616
*P. antarctica*	Antarctica, Livingston Island, Byers Peninsula. S 62°40.144′, W 61°10.333′; 70 m asl./LI-By-W	>5000 a	M-0019670/ Byer 15/01aII	B1615
*P. antarctica*	Antarctica, Robert Island, Coppermine Peninsula. S 62°22,311′, W 59°43.024′; 150 m asl./RI-Co	>5000 a	M-0019707/ Robe 15/01cVI	B1665
*P. antarctica*	Antarctica, Robert Island, Coppermine Peninsula. S 62°22,311′, W 59°43.024′; 150 m asl./RI-Co	>5000 a	M-0019669/ Robe 15/01aII	B1614
*P. antarctica*	Antarctica, King George Island, Fildes Peninsula. S 62°12.243′, W 58°57.590′; ca. 70 m asl./KGI-Fi-C	>5000 a	M-0019729/ 140109_MCRU	B1597
*P.* *contortuplicata*	Antarctica, Deception Island. S 62°56.330′, W 60°35.648′; 30 m asl./DI-NE		M-0019705/ Dec 15/08aVI	B1663
*P.* *contortuplicata*	Antarctica, King George Island, Fildes Peninsula. S 62°12.360′, W 58°57.239′; 49 m asl./KGI-Fi-C	>5000 a	M-0019657/ MCRU 15/01cII	B1602
*P.* *contortuplicata*	Antarctica, King George Island, Fildes Peninsula. S 62°12.360′, W 58°57.239′; 49 m asl./KGI-Fi-C	>5000 a	M-0019679/ MCRU 15/01bV	B1624
*P.* *contortuplicata*	Antarctica, King George Island. Northern part of Fildes Peninsula. S 62°11.780′, W 58°59.602′; 33 m asl./KGI-Fi-NW	<5000 a	M-0019660/ VALK 15/01cVI	B1605
*P.* *contortuplicata*	Antarctica, King George Island. Northern part of Fildes Peninsula. S 62°11.780′, W 58°59.602′; 33 m asl./KGI-Fi-NW	<5000 a	M-0019680/ VALK 15/01aIII	B1625
*P.* *contortuplicata*	Antarctica, Livingston Island, Byers Peninsula. S 62°40.065′, W 60°54.739′; 130 m asl./LI-By-E	>5000 a	M-0019683/ Byer 15/06fIII	B1628
*P.* *contortuplicata*	Antarctica, Livingston Island, Byers Peninsula. S 62°40.220′, W 61°06.776′; 73 m asl./LI-By-C	>5000 a	M-0019677/ 140127d_ByCeCol	B1622
*P.* *contortuplicata*	Antarctica, King George Island. Northern part of Fildes Peninsula. S 62°11.780′, W 58°59.602′; 33 m asl./KGI-Fi-NW	<5000 a	M-0019681/ VALK 15/01eI	B1626
*Ramalina terebrata*	Antarctica, Deception Island. S 62°59.272′, W 60°33.006′; 59 m asl.		M-0019724/140124e	B1592
*R. terebrata*	Antarctica, King George Island, Ardley. S 62°12.675′, W 58°55.461′; 18 m asl.	>5000 a	M-0019728/140116d	B1596
*R. terebrata*	Antarctica, King George Island, Ardley. S 62°12.55′, W 58°56.416′; 25 m asl.	>5000 a	M-0019727/140116f	B1595
*R. terebrata*	Antarctica, King George Island. Fildes Peninsula, Nebles Point. S 62°11.033′, W 58°51.544′; 9 m asl.	>5000 a	M-0019725/140118c	B1593
*R. terebrata*	Antarctica, King George Island, Potter Peninsula. S 62°14.834′, W 58°40.760′; 20 m asl.	>5000 a	M-0019726/140117b2	B1594
*R. terebrata*	Antarctica, Livingston Island, Byers peninsula, Playa los Presidentes. S 62°39.325′, W 61°08.792′; 2 m asl.	>5000 a	M-0019722/140130d	B1590
*R. terebrata*	Antarctica, Livingston Island, Byers peninsula, Punta Petreles. S 62°40.496′, W 61°05.807′; 13 m asl.	>5000 a	M-0019723/140127a	B1591

## Data Availability

Data is in Supplementary Materials.

## Supplementary Materials

The following supporting information can be downloaded at: https://www.mdpi.com/article/10.3390/genes14051019/s1, The details involved in the article can be found in Supplementary Data Table S1 and Supplementary Materials Scripts.
