# Supplementary material for: Metabarcoding of Antarctic Lichens from Areas with Different Deglaciation Times Reveals a High Diversity of Lichen-Associated Communities
Source: Genes (Basel). 2023 Apr 29;14(5):1019. doi: 10.3390/genes14051019 (PMC10218603; doi:10.3390/genes14051019)
Supplement: Supplementary file 1 [file genes-14-01019-s001.zip › Beck&al2023_SuplMat_Scripts.pdf]

Beck et al. 2023: Metabarcoding of Antarctic lichens from areas with different deglaciation times reveals a high diversity of lichen-associated communities

Scripts used for diversity estimations

Coverage-based rarefaction/extrapolation

```
library(iNEXT)
library(writexl)

read.csv("metabarcoding_less_5000.csv", row.names = 1, header = TRUE) ->
meta_less
read.csv("metabarcoding_more_5000.csv", row.names = 1, header = TRUE) ->
meta_more
read.csv("metabarcoding_deception.csv", row.names = 1, header = TRUE) ->
meta_dec

list1 = t(meta_more) # transposed list to have ASVs in columns and
samples in rows
list2 = t(meta_less) # transposed list to have ASVs in columns and
samples in rows
list3 = t(meta_dec) # transposed list to have ASVs in columns and samples
in rows
list(list1,list2,list3) -> combined_lists
# gives names to the lists
names(combined_lists) <- c("Deglaciation > 5000 yr", "Deglaciation < 5000
yr", "Deception Island")
out <- iNEXT(combined_lists, q=1, datatype = "incidence_raw", conf =
0.95, se=T) # se option for showing confidence interval in the plot
SC_list <- out$iNextEst$coverage_based # to explore sample coverage
values
write.csv(SC_list,"SC_coverage.csv")
tiff('coverage_q_2_size_1.0_conf.tiff', units="in", width=15, height=10,
res=300, compression = 'lzw')
ggiNEXT(out, type=3) + # type 3 data set calculates coverage based
rarefaction/extrapolation plot
  xlim(c(0.4,1.0)) + # xlim limits from/to which % coverage it shows on
the plot
  theme_bw(base_size = 18) + # size of the figure
  theme_light() +
  labs(x = "Coverage", y = "Diversity") +
  theme(axis.text = element_text(size = 20)) +
  theme(axis.title = element_text(size = 20)) +
  theme(legend.text = element_text(size = 17)) +
  theme(legend.position="bottom")
dev.off()
```

Sample-size-based rarefaction/extrapolation

```
library(iNEXT)
library(writexl)
```

```
read.csv("metabarcoding_less_5000.csv", row.names = 1, header = TRUE) ->
meta_less
read.csv("metabarcoding_more_5000.csv", row.names = 1, header = TRUE) ->
meta_more
read.csv("metabarcoding_deception.csv", row.names = 1, header = TRUE) ->
meta_dec
list1 = t(meta_more) # transposed list to have ASVs in columns and
samples in rows
list2 = t(meta_less) # transposed list to have ASVs in columns and
samples in rows
list3 = t(meta_dec) # transposed list to have ASVs in columns and samples
in rows
list(list1,list2,list3) -> combined_lists
# gives names to the lists
names(combined_lists) <- c("Deglaciation > 5000 yr", "Deglaciation < 5000
yr", "Deception Island")
out <- iNEXT(combined_lists, q=2, endpoint=32, datatype =
"incidence_raw", conf = 0.95, se=FALSE)
tiff('interpolation_extrapolation_size_m_q_2.tiff', units="in", width=15,
height=10, res=300, compression = 'lzw')
ggiNEXT(out) +
  theme_light() +
  labs(x = "Number of sampling units", y = "Diversity") +
  theme(axis.text = element_text(size = 20)) +
  theme(axis.title = element_text(size = 20)) +
  theme(legend.text = element_text(size = 17)) +
  theme(legend.position="bottom")
dev.off()
```

Alpha diversity calculations

```
library(vegan)
library(ggplot2)
library(agricolae)

#with example data
read.csv("metabarcoding_abundance_more5000.csv", row.names = 1, header =
TRUE) -> more_abundance
Deglaciation_time = c("more_than_5000_ya") #add column Deglaciation time
with more than 5000 ya
more_abundance <- cbind(more_abundance, Deglaciation_time)
read.csv("metabarcoding_abundance_less5000.csv", row.names = 1, header =
TRUE) -> less_abundance
Deglaciation_time = c("less_than_5000_ya") #add column Deglaciation time
with less than 5000 ya
less_abundance <- cbind(less_abundance, Deglaciation_time)
read.csv("metabarcoding_abundance_dec.csv", row.names = 1, header = TRUE)
-> dec_abundance
Deglaciation_time = c("Deception_Island") #add column Deglaciation time
with Deception island
dec_abundance <- cbind(dec_abundance, Deglaciation_time)
binded_abundance_all <- bind_rows(more_abundance,less_abundance) %>% #
binds all three data frames in one
  bind_rows(dec_abundance)
# calculates a shannon index, excluding the last column with Deglaciation
time info
binded_abundance_all$alpha <- diversity(binded_abundance_all[,-130],
index = "shannon")
```

```
tiff("alpha_diversity_plot.tiff", units="in", width=5, height=5, res=300,
compression = 'lzw') # save as tiff
ggplot(binded_abundance_all, aes(x = Deglaciation_time, y = alpha)) +
  geom_boxplot(outlier.shape = NA) +
  geom_jitter(width = 0.2, colour=2) +
  theme_light() +
  theme(axis.text = element_text(size = 20)) +
  theme(axis.title = element_text(size = 20))
dev.off()

# calculate for a significance of the diversity difference
anova_result <- aov(alpha ~ Deglaciation_time, binded_abundance_all)
summary(anova_result)
anova_HSD_test <- HSD.test(anova_result, "Deglaciation_time", alpha =
0.01)
print(anova_HSD_test)
```
